# Cutaneous Anaplastic Large-Cell Lymphoma with Dramatic Response to Brentuximab Vedotin

**DOI:** 10.4274/tjh.galenos.2020.2020.0512

**Published:** 2021-02-25

**Authors:** Mustafa Şahin, Mine Miskioğlu, Işıl Inanır, Hikmet Akar, Nalan Neşe, Peyker Temiz, İsmet Aydoğdu

**Affiliations:** 1 Celal Bayar University Faculty of Medicine, Department of Internal Medicine, Manisa, Turkey; 2 Celal Bayar University Faculty of Medicine, Department of Hematology, Manisa, Turkey; 3 Celal Bayar University Faculty of Medicine, Department of Dermatology, Manisa, Turkey; 4 Celal Bayar University Faculty of Medicine, Department of Patology, Manisa, Turkey

**Keywords:** Cutaneous anaplastic large cell lymphoma, Brentuximab, Monotherapy, Breast cancer

## To the Editor,

Cutaneous lymphomas account for 5% of non-Hodgkin lymphomas (NHLs). With 75% of skin lymphomas originating from T-cells, CD30+ anaplastic large-cell NHL (ALCL) is the second most common type after mycosis fungoides. In treatment, excision and radiotherapy are the first options for single lesions, but systemic treatment is required for multiple ones. Brentuximab vedotin (BV) is an antibody-drug conjugate composed of a CD30-directed monoclonal antibody and monomethyl auristatin E. Recently, it has been used in the treatment of Hodgkin lymphoma and chemotherapy-resistant ALCL [1,2,3].

An 84-year-old woman presented to the dermatology department with an erythematous plaque below the right eye, which had been present for 3 months. A round and firm nodule of 5 cm was also observed on the left breast. The mammography was consistent with a BI-RADS 5 lesion. The histopathological diagnoses were cutaneous ALCL for the facial lesions and invasive ductal carcinoma for the breast nodule. She declined treatment for both.

**Figure 1:**
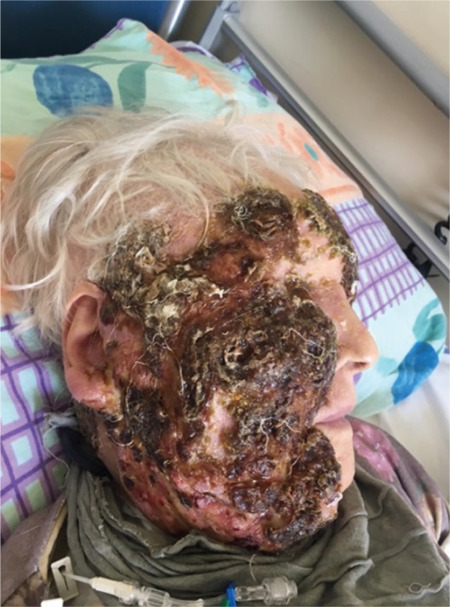
Before treatment.

Three months thereafter, she was admitted with an ulcerative infiltration of the entire right side of the face, invading the right side of the frontal area (Figure 1). She also had weakness and loss of appetite. Hematological and biochemical parameters including lactate dehydrogenase were normal other than hemoglobin of 11.2 g/dL, and bone marrow biopsy results were normocellular. positron emission tomography-computed tomography (PET/CT) showed involvement of the right side of the face, left breast, and right cervical and mediastinal lymph nodes, with millimetric skin lesions of the whole body, especially in the right femur. The involvements other than the face were thought to be related to breast cancer, but this could not be confirmed by biopsy. The patient again did not accept surgery or other treatments for invasive ductal carcinoma.

**Figure 2:**
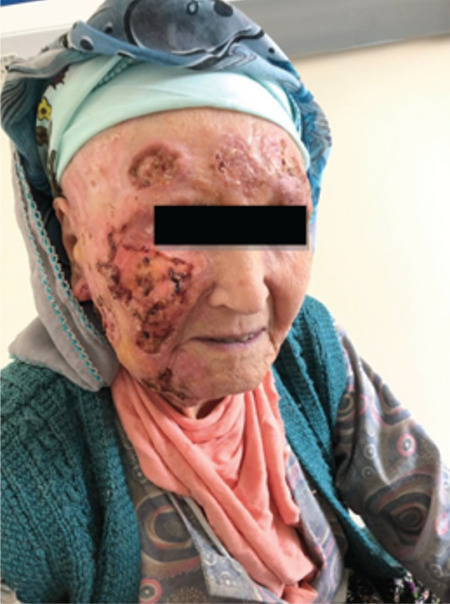
After the first cycle.

**Figure 3:**
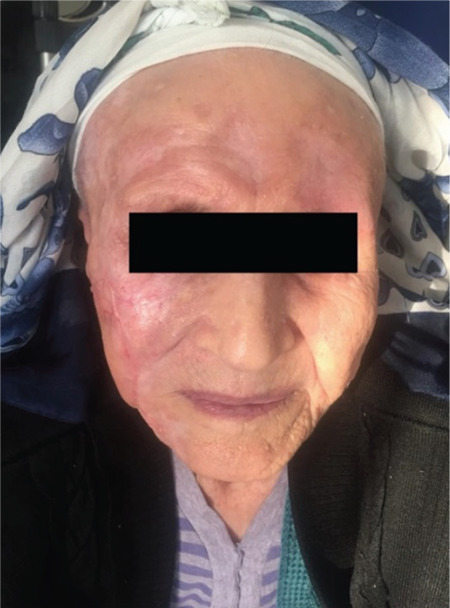
After the fourth cycle.

Due to advanced age and frailty, combination chemotherapy was considered unsuitable, so BV monotherapy was administered at a dose of 1.8 mg/kg every 21 days for four cycles. Anastrozole was also added according to the recommendations of medical oncology. The facial lesions regressed significantly after the first cycle and disappeared after the fourth one, and up to 50% reduction in the breast mass was observed. In PET/CT, although the involvement of the cervical, supraclavicular, and thoracic lymph nodes continued, the lesions in the skin, breast, and femur had disappeared. These findings suggested primary cutaneous ALCL, but it is difficult to draw a definitive conclusion. It is thought that the regression in the breast lesion might be related to the anastrozole taken with brentuximab. There was no BV-related toxicity and daily activities improved (Figures 2 and 3).

There are several reports presenting the effectiveness of BV treatment for ALCL [4,5,6]. The ECHELON-2 (a BV-containing regimen) and ALCANZA (BV alone) studies showed higher rates of progression-free survival than regimens without BV for CD30+ cutaneous T-cell lymphoma patients [5,6]. As in our case, it may be a good option to use BV as first-line monotherapy for cases not suitable for combined chemotherapy [1,2,3].

## References

[1] O’Connor OA, Marchi E, Bhagat G, Coradini P, Guitart J, Rosen ST (2013.). T-cell lymphomas. In: Hoffman R, Benz EJ Jr, Silberstein LE, Heslop HE, Weitz JI, Anastasi J (eds). Hematology Basic Principles and Practice (6th Edition). Philadelphia, Elsevier Saunders.

[2] Willemze R, Hodak E, Zinzani PL, Specht L, Ladetto M;, ESMO Guidelines Working Group (2013). Primary cutaneous lymphomas: ESMO Clinical Practice Guidelines for diagnosis, treatment and follow-up. Ann Oncol.

[3] Pro B, Advani R, Brice P, Bartlett NL, Rosenblatt JD, Illidge T, Matous J, Ramchandren R, Fanale M, Connors JM, Yang Y, Sievers EL, Kennedy DA, Shustov A (2012). Brentuximab vedotin (SGN-35) in patients with relapsed or refractory systemic anaplastic large-cell lymphoma: results of a phase II study. J Clin Oncol.

[4] Onaka T, Kitagawa T, Kawakami C, Yonezawa A (2018). Improvement of cutaneous anaplastic large cell lymphoma by brentuximab vedotin monotherapy. Turk J Hematol.

[5] Horwitz S, O’Connor OA, Pro B, Illidge T, Fanale M, Advani R, Bartlett NL, Christensen JH, Morschhauser F, Domingo-Domenech E, Rossi G, Kim WS, Feldman T, Lennard A, Belada D, Illés Á, Tobinai K, Tsukasaki K, Yeh SP, Shustov A, Hüttmann A, Savage KJ, Yuen S, Iyer S, Zinzani PL, Hua Z, Little M, Rao S, Woolery J, Manley T, Trümper L;, ECHELON-2 Study Group (2019). Brentuximab vedotin with chemotherapy for CD30-positive peripheral T-cell lymphoma (ECHELON-2): a global, double-blind, randomised, phase 3 trial. Lancet.

[6] Prince HM, Kim YH, Horwitz SM, Dummer R, Scarisbrick J, Quaglino P, Zinzani PL, Wolter P, Sanches JA, Ortiz-Romero PL, Akilov OE, Geskin L, Trotman J, Taylor K, Dalle S, Weichenthal M, Walewski J, Fisher D, Dréno B, Stadler R, Feldman T, Kuzel TM, Wang Y, Palanca-Wessels MC, Zagadailov E, Trepicchio WL, Zhang W, Lin HM, Liu Y, Huebner D, Little M, Whittaker S, Duvic M;, ALCANZA Study Group (2017). Brentuximab vedotin or physician’s choice in CD30-positive cutaneous T-cell lymphoma (ALCANZA): an international, open-label, randomised, phase 3, multicentre trial. Lancet.

